# Cervicofacial Actinomycosis Presenting as a Fistulized Neck Mass During Pregnancy: A Case Report

**DOI:** 10.3390/diagnostics16081140

**Published:** 2026-04-10

**Authors:** Florentina Severin, Andrei Alexandru Andoni, Raluca Ioana Serban, Andrei Nicolau, Deniss Vasile Mereuta, Andreea Vlad, Florin Mocanu, Ionut Andrei Roman, Octavian Dragos Palade

**Affiliations:** 1 ENT Department, Faculty of Medicine, “Grigore T. Popa” University of Medicine and Pharmacy, 700115 Iasi, Romaniavlad.andreea@email.umfiasi.ro (A.V.); florin.mocanu@umfiasi.ro (F.M.);; 2 Surgery Department, Faculty of Medicine, “Grigore T. Popa” University of Medicine and Pharmacy, 700115 Iasi, Romania; 3 Microbiology Department, Faculty of Medicine, “Grigore T. Popa” University of Medicine and Pharmacy, 700115 Iasi, Romania; 4 Oral and Maxillo-Facial Surgery Department, “Grigore T. Popa” University of Medicine and Pharmacy, 700115 Iasi, Romania; 5 Anatomy Department, Faculty of Medicine, “Grigore T. Popa” University of Medicine and Pharmacy, 700115 Iasi, Romania; deniss.mereuta@umfiasi.ro

**Keywords:** actinomycosis, fistulized neck mass, pregnancy, multidisciplinary approach, microbiological assays

## Abstract

**Background**: Cervicofacial actinomycosis is an uncommon chronic bacterial infection that can mimic neoplasia or granulomatous disease because of its infiltrative presentation. Diagnosis is often delayed, particularly in pregnant patients in whom imaging and invasive procedures may be limited. **Case report**: A 25-year-old woman at 14 weeks of gestation presented with a multiple-fistulized cervical mass. The lesion was initially diagnosed as a cutaneous furuncle in a private dermatology practice and treated with topical therapy, resulting in only transient improvement. Two weeks later, multiple fistulizations developed, prompting consultation in the emergency department. ENT assessment and ultrasound raised suspicion of cervical actinomycosis versus fistulized tuberculous lymphadenitis. Considering the pregnancy, drainage of the collection was performed under local anesthesia and empiric antibiotic therapy with amoxicilin-clavulanic acid was started. Microbiological confirmation of *Actinomyces (Schaalia) georgiae* led to infectious disease evaluation that established a long-term antibiotic therapy while monitoring fetal safety. Progressive clinical improvement was observed, with complete resolution after three months. The pregnancy progressed without complications and fetal morphology remained normal under therapy. **Conclusions**: This case illustrates the diagnostic complexity of cervicofacial actinomycosis caused by *A. georgiae* during pregnancy, representing the first such report in the current literature, and emphasizes the need for a multidisciplinary approach.

## 1. Introduction

Cervicofacial actinomycosis, also known as ‘lumpy jaw syndrome’, is an infection caused by anaerobic Gram-positive filamentous bacilli of the *Actinomyces* species and related organisms, some of which have been reclassified following recent taxonomic revisions, which are found in the commensal flora of the oral cavity, in gingival crevices, periodontal pockets, tonsillar crypts, as well as on carious teeth and in dental plaque [1]. *Actinomyces* and related organisms can also be found in other anatomical sites, including the skin and the gastrointestinal and genitourinary tracts. *Actinomyces israelii* has long been recognized as a causative agent of actinomycosis, but 35 different *Actinomyces* species have been described [2] and more than 26 species are associated with humans, including *Actinomyces odontolyticus* (now classified within the genus *Schaalia*), *Actinomyces naeslundii*, *Actinomyces meyeri*, *Actinomyces viscosus*, *Actinomyces funkei*, *Actinomyces gerencseriae*, *Actinomyces urogenitalis*, *Actinomyces georgiae* (now classified within the genus *Schaalia*), and *Actinomyces graevenitzii*. Notably, several of these organisms have been reclassified due to the recent phylogenetic taxonomic revisions; as an example, *Actinomyces pyogenes* has been reclassified as *Trueperella pyogenes* [3]. Diagnosis may be difficult because of a general lack of familiarity with the disease and a low success rate in culturing the organism as a result of its fastidious nature. As the cervicofacial manifestations of actinomycosis are varied, a high index of suspicion is required for an accurate and timely diagnosis [4]. Infections may present in acute, subacute, or chronic forms, often following trauma or dental infection. The clinical presentation may mimic other conditions, such as solid tumors, tuberculosis, nocardiosis, or fungal infections, and is often characterized by painful subcutaneous induration, purplish discoloration of the overlying skin, and regional lymphadenopathy. The appearance of cutaneous fistulas, from which thick pus containing yellowish granules (sulfur granules) drains spontaneously, is characteristic. The most frequent localization is in the submandibular region. A distinctive sign of cervicofacial actinomycosis is the tendency to spread without regard for anatomical barriers, including fascial planes or lymphatic drainage, and the development of multiple sinus tracts [5]. Laboratory confirmation of the diagnosis requires drainage with Gram staining, bacteriological culture under anaerobic conditions, cytological evaluation of the recovered fluid, and/or histopathological examination demonstrating the characteristic sulfur granules.

Diagnostic imaging technologies such as CT and MRI usually deliver nonspecific findings, contributing only to define radiological features of the mass and its involvement in adjacent soft tissues. Treatment consists of long-term antibiotic therapy and drainage of abscesses [6]. It is mandatory to customize the treatment based on the anatomical region involved, extension, and response to the antibiotic therapy.

Actinomycosis occurring during pregnancy is exceptional. Reported cases are mainly associated with chorioamnionitis and an increased risk of preterm birth [7]. To date, no cervicofacial presentations attributed to *A. georgiae* have been documented in pregnant patients.

We report a rare case of cervical actinomycosis occurring during pregnancy and discuss the diagnostic and therapeutic challenges. The significance of this particular case is primarily represented by the location of the fistulized neck mass in the left upper laterocervical region, which leads to several differential diagnoses for the clinician and contributes significantly to the existing literature by drawing attention to this location and avoiding diagnostic errors. This adds valuable insights to the diverse manifestations of actinomycosis in pregnancy, potentially influencing diagnostic protocols and therapeutic approaches specific to cases involving the neck region.

## 2. Case Report

**Figure 1:**
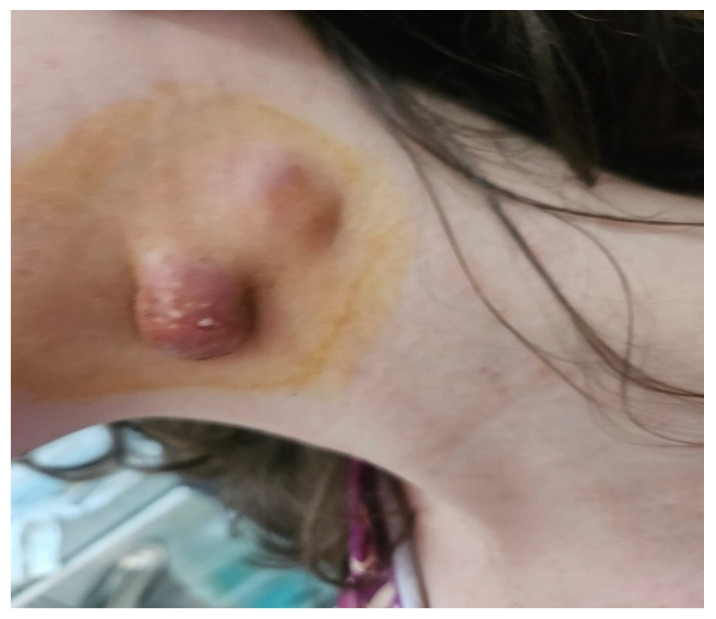
Clinical aspect of the fistulized cervical mass.

A 25-year-old woman at 14 weeks of gestation, with no significant past medical history, presented at the end of November 2025 to the Emergency Department of St. Spiridon Emergency Clinical Hospital, Iasi, Romania, for evaluation of a fistulized cervical mass (Figure 1).

She was subsequently referred for an ENT consultation. The patient reported that the swelling had been slowly enlarging over the previous four months without inflammatory signs until two weeks prior to presentation. At the time, at the beginning of November 2025, she had been diagnosed with a cutaneous furuncle in a private dermatology practice and treated with topical therapy (neomycin sulfate and prednisolone ointment), with only temporary improvement. Shortly thereafter, multiple fistulous tracts developed. She denied any history of chronic disease, recent dental infection, or cervicofacial trauma. The patient also reported no fever, chills, weight loss, or other constitutional symptoms, and had no known history of tuberculosis.

On physical examination, the patient had good oral hygiene and no evidence of poor dentition. The patient denied smoking, alcohol consumption, recent dental procedures, or oral trauma.

To evaluate the fistulized neck mass located in the left upper laterocervical region, a clinical examination associated with ultrasound was performed. The mass was covered by erythematous skin with multiple fistulae, some of which were draining purulent material. Clinical examination detected an oval, fixed and painful tumefaction with a diameter of 28/15/10 mm, with a elastic-fluctuent consistency. A clinical diagnostic assumption was made: fistulized cervicofacial actinomycosis vs. fistulized ganglionic tuberculosis (scrofuloderma).

**Figure 2:**
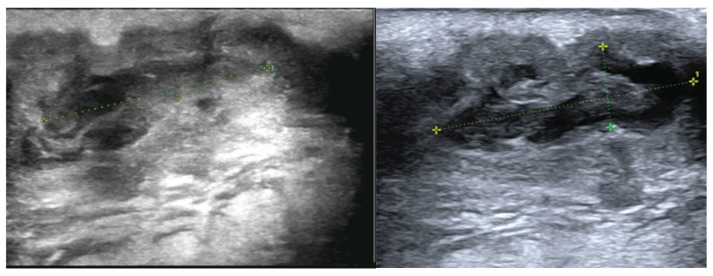
Ultrasonographic images of the cervical mass.

Ultrasonographic assessment was limited because the lesion was highly painful on palpation. A 20 × 8 × 8 mm heterogeneous hypoechoic lesion with a preserved vascular hilum and marked internal vascularity was identified. Several intralesional hyperechoic nodules measuring 2–3 mm were noted. The lesion extended deeply through a millimetric fistulous tract toward a second, similar collection measuring 30 × 7 mm, which further communicated posteriorly with another superficial, palpable fistulous focus measuring 10 × 5 mm. The initially described lesion was located superficially to and in close contact with the submandibular gland. No deep cervical lymphadenopathy was identified (Figure 2).

Laboratory investigations revealed mild anemia (hemoglobin 11.8 g/dL; hematocrit 34.8%). Mild elevations in bilirubin levels were also observed (total bilirubin 1.26 mg/dL; direct bilirubin 0.37 mg/dL), while the remaining biochemical parameters were within normal limits (Table 1).

No further imagistic tests were made. In pregnancy, ultrasound and magnetic resonance imaging are generally preferred; however, X-ray and computed tomography may be performed when medically necessary for appropriate clinical management and when other imaging modalities are unavailable or insufficient for diagnosis. Emergency MRI of the neck was not available in our hospital.

Following the clinical and ultrasound examination, we decided to admit the patient in the ENT clinic for surgical drainage of the mass under local anesthesia due to the pregnancy risk. The patient declined inpatient admission and agreed with the surgical treatment in an outpatient system, after the patient provided written informed consent. An incision was made on the area of maximum fluctuation and fistulization of the neck mass, we identified a purulent secretion with granular yellowish debris, which after the drainage left a cavity with granular bleeding walls. Although the presence of granular material could have prompted immediate microscopic examination (e.g., crushed preparation or direct Gram stain), this was not performed intraoperatively. Given the clinical presentation, tuberculosis remained an important differential diagnosis, and further investigations, including MTB PCR, were pursued. Several specimens were collected: two aspirated fluids and one pus sample obtained by curettage. Based on the clinical history and physical examination, several conditions were considered in the differential diagnosis, including tumoral, granulomatous, and other suppurative diseases. Therefore, samples from the lesion were collected for direct microscopy, *Mycobacterium tuberculosis* DNA detection, and bacteriological culture (including anaerobic culture). Mycobacterial (AFB) cultures were performed, but remained negative. MTB PCR was undertaken to allow rapid differentiation in the context of suspected tuberculosis. Following the establishment of the diagnosis of cervicofacial actinomycosis, further evaluation for tuberculosis lymphadenitis was deemed unnecessary. Surgical debridement and wound toilet were performed, and the incision was left open with placement of cotton gauze followed by local dressings. Daily wound care was continued on an outpatient basis in the ENT clinic. Empiric antibiotic therapy with amoxicillin-clavulanic acid (1 g twice a day) was initiated. Additional evaluations by the pulmonology, infectious diseases, and obstetrics–gynecology teams were requested. The pulmonology team ruled out active pulmonary tuberculosis based on clinical evaluation and sputum examination, which showed no features suggestive of active disease.

**Figure 3:**
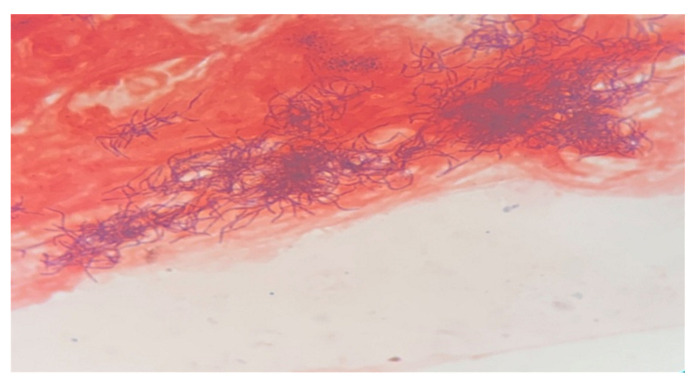
Smear from purulent material, Gram stain (PMNs, Gram-positive bacilli).

**Figure 4:**
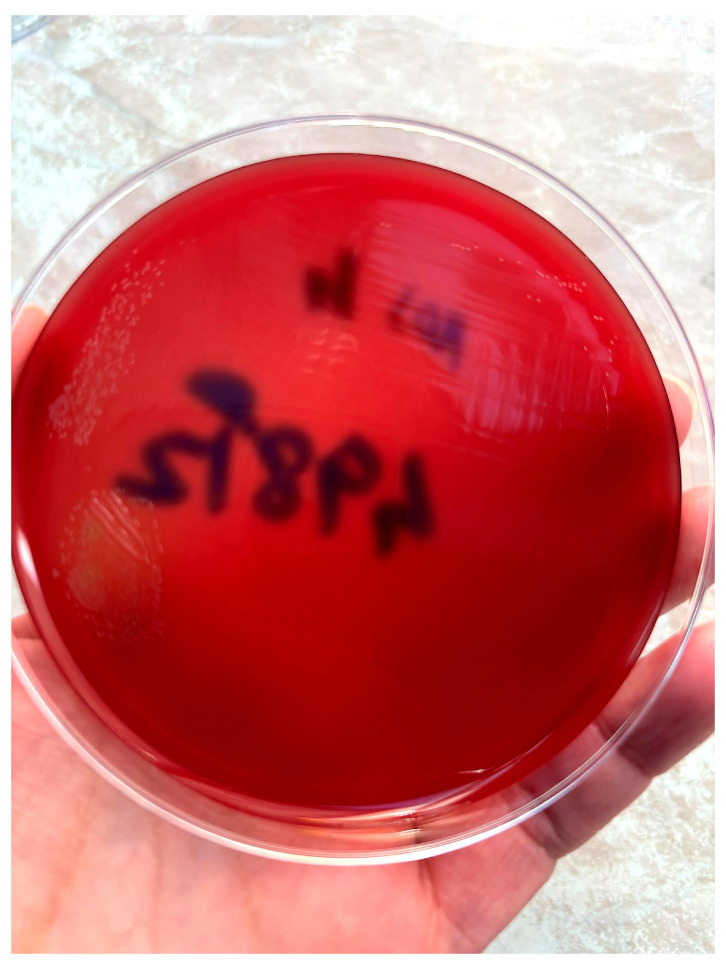
*Actinomyces georgiae*, culture on Columbia agar.

Molecular testing by PCR (ELITe InGenius) for Mycobacterium tuberculosis was negative. Molecular testing was performed as part of the initial evaluation for tuberculous lymphadenitis; however, given its lower sensitivity in extrapulmonary specimens, a negative result alone cannot definitively exclude this diagnosis. The specimen had a purulent and hemorrhagic appearance. Gram-stained smears were prepared from each sample. Microscopic examination revealed a marked inflammatory reaction in only one smear (from the purulent material). Actinomycosis is typically a polymicrobial anaerobic infection. The identification of filamentous, branching Gram-positive rods with a beaded appearance on Gram staining, together with a negative modified acid-fast bacilli (AFB) stain, supports the diagnosis of *Actinomyces* infection and helps differentiate it from *Nocardia* species (Figure 3). The presumptive diagnosis was essential for further patient management and follow-up. Cultures on solid media remained negative after 48 h. Because no growth was observed after the initial incubation period, the enrichment broths (thioglycolate) were maintained for further incubation. Subsequently, *Actinomyces georgiae* was isolated from the enrichment broth and identified by MALDI-TOF mass spectrometry using the Bruker MALDI Biotyper (Bruker Corporation, Bremen, Germany) system with the manufacturer’s reference spectral library, after subculture onto Columbia agar supplemented with 5% sheep blood and incubated under anaerobic conditions for 4 days (Figure 4).

After subculture onto Columbia agar and incubation under anaerobic conditions for 4 days, small whitish colonies with a rough surface were observed.

Cytopathological evaluation of the aspirated purulent material stained with hematoxylin–eosin revealed round basophilic aggregates composed of filamentous bacteria surrounded by an eosinophilic border, consistent with the Splendore–Hoeppli phenomenon.

Following microbiological confirmation of *Actinomyces georgiae*, the infectious diseases consultation was obtained to establish prolonged antibiotic therapy and to evaluate the potential risks to the ongoing pregnancy. Amoxicillin-clavulanic acid was prescribed for 3 months. During the course of treatment, the patient underwent obstetric–gynecological consultation and fetal morphological evaluation to monitor the pregnancy. The therapeutic strategy was carefully tailored to the patient’s physiological state. Amoxicillin-clavulanic acid was selected as the primary agent due to its broad-spectrum efficacy against *Actinomyces* species and frequent co-pathogens, as well as its well-documented safety profile during pregnancy. While traditional regimens for actinomycosis often extend from 6 to 12 months, the 3-month duration in this case was justified by the prompt clinical response following surgical drainage and the complete closure of all fistula tracts. To ensure sustained resolution, the patient was monitored for 6 months post-therapy, with no signs of recurrence.

**Figure 5:**
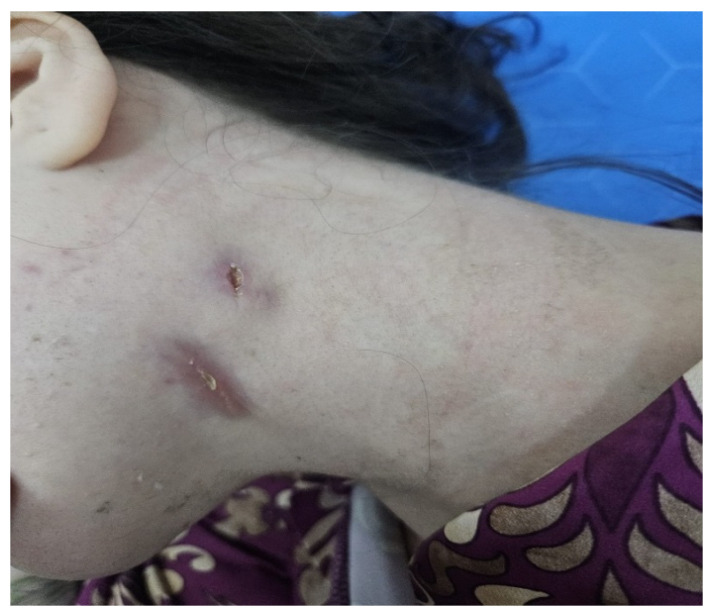
Appearance of the surgical wound at 1 month postoperatively.

The surgical drainage site required daily toileting for 1 month with slowly progressive healing, without residual fistula tracts (Figure 5). Complete clinical resolution was observed, with no recurrence during follow-up.

## 3. Discussion

The classification of actinomycosis as a neglected disease stems from its relatively low incidence, which has resulted in a lack of systematic global monitoring. Currently, the WHO does not maintain a dedicated longitudinal database to track the global burden of the infection [2].

*Actinomyces* species are anaerobic, non-spore-forming, Gram-positive rods characterized by a radial arrangement of bacterial filaments. Taxonomically, the genus *Actinomyces* belongs to the order *Actinomycetales* within the *phylum Actinobacteria* [2].

*Actinomyces* species are commensal organisms that normally inhabit the oral cavity and other mucosal surfaces of humans, appearing in significant concentrations within the gingival and periodontal spaces, as well as the crypts of the palatine tonsils [8]. Numerous *Actinomyces* species have been isolated from soft tissue abscesses localized to the upper anatomical regions. These suppurative lesions frequently involve the cervicofacial area, the axillary vaults, the thorax, and the posterior trunk, with *A. europaeus*, *A. georgiae*, *A. meyeri*, *A. neuii* (both subspecies), and *A. radingae* being the identified species [9]. Notably, while *A. israelii* is recognized as the primary etiological agent of human actinomycosis, it is infrequently detected within the oral microbiome of pediatric populations. Conversely, *A. georgiae* appears to be more prevalent in this demographic, with the majority of characterized isolates originating from the gingival sulci of periodontally healthy children [10].

Actinomycosis is a slowly progressive granulomatous infection with a male predilection, affecting individuals regardless of immune status. It typically presents in three primary clinical forms: cervicofacial, thoracic, and abdominopelvic. The diagnostic process is frequently complicated by the nonspecific nature of initial symptoms. Key clinical features of advanced disease include chronic inflammatory manifestations, the formation of abscesses with associated sinus tracts, and the discharge of purulent exudate [11].

While actinomycosis is a well-recognized chronic granulomatous infection, its diagnosis has traditionally been predicated on clinical observation and histopathology due to infrequent microbiological confirmation. False-negative culture results are relatively common in actinomycosis and are often related to the difficulty of isolating slow-growing anaerobic organisms in clinical laboratories. Recovery of *Actinomyces* species may be challenging, particularly in polymicrobial infections where other bacteria may overgrow the culture. Inadequate anaerobic conditions or insufficient incubation time may also contribute to negative culture results, while prior antibiotic therapy may play a lesser role unless prolonged treatment has been administered. Although molecular methods have been explored for the detection of *Actinomyces* species, their use in routine clinical practice remains limited. In non-sterile specimens, molecular testing may detect DNA from commensal organisms, which can complicate interpretation. Therefore, histocytopathological examination demonstrating characteristic sulfur granules and filamentous bacteria remains one of the most reliable methods for confirming the diagnosis of actinomycosis [12].

The cervicofacial subtype constitutes over half of all actinomycosis presentations, making it the most frequent clinical form. Its etiology is closely linked to poor oral hygiene and lifestyle factors such as smoking and heavy alcohol use. Additionally, invasive dental procedures, particularly extractions, are recognized as critical predisposing elements [13]. The etiological agent consisting primarily of *A. israelii* (42%) and *A. gerencseriae* (26.7%). In addition, *A. naeslundii*/”*A. viscosus*” was found in ~9% of the specimens, while *A. odontolyticus*, *A. meyeri*, *A. georgiae*, and *A. neuii* subsp. were occasionally recovered [14]. In the literature, few cases of actinomycosis in pregnancy have been reported, but none with *Actinomyces georgiae* [15].

Actinomycosis in pregnancy is rare, and the species involved vary depending on the anatomical site of infection. Reported cases have most commonly involved *Actinomyces israelii,* which is the classical pathogen associated with cervicofacial actinomycosis. Other species described in the literature include *Actinomyces naeslundii*, *Actinomyces odontolyticus*, *Actinomyces turicensis*, and *Actinomyces meyeri*. These infections typically present as cervicofacial abscesses, mandibular swelling, draining sinus tracts, or odontogenic infections.

The pathogenesis of actinomycosis is initiated by a breach in the mucocutaneous barrier, which facilitates suppurative inflammation and subsequent abscess formation. Contiguous tissue invasion remains the primary mechanism of dissemination. While hematogenous spread to distant sites—most notably the central nervous system and pulmonary parenchyma—is well-documented, lymphatic involvement is considered an atypical finding [16].

In suspected cases of cervicofacial actinomycosis, the diagnostic protocol should involve a microscopic examination of clinical specimens to identify pathognomonic sulfur granules and dense aggregates of branching Gram-positive filaments. This approach, which was employed in the present case, is increasingly complemented by matrix-assisted laser desorption/ionization time-of-flight (MALDI-TOF) mass spectrometry, a technology widely used in clinical microbiology laboratories for rapid and accurate identification of bacterial isolates after growth on culture media [17]. In a comparative study, MALDI-TOF mass spectrometry correctly identified 97% of 32 strains to the species level, while a commercially available biochemical kit achieved only 33% success [18]. Microbiological evaluation remains pivotal for a definitive diagnosis; however, isolation of Actinomyces species can be challenging because these organisms are fastidious and often grow slowly under anaerobic or microaerophilic conditions. The necessity for prolonged incubation—extending up to 14 days under strict anaerobic conditions—together with overgrowth by other anaerobic bacteria in polymicrobial cultures and the suppressive effects of antecedent antimicrobial therapy, significantly complicates the diagnostic yield of conventional cultures [19].

Accurate diagnostic identification is a critical determinant of clinical management strategies and subsequent patient outcomes. Actinomycosis typically presents as a suppurative granulomatous infection characterized by microabscess formation and inflammatory infiltrates composed predominantly of neutrophils and macrophages. In aspirated purulent material, cytopathological examination may reveal aggregates of filamentous bacteria consistent with sulfur granules, which support the diagnosis [16].

The clinical diagnosis of cervicofacial actinomycosis is initially challenging and requires a high index of suspicion; however, the presence of pathognomonic sulfur granules makes the clinical presentation characteristic of cervicofacial actinomycosis. The differential diagnosis encompasses a broad spectrum of inflammatory, infectious, and neoplastic conditions. Pyogenic abscesses and fistulae were considered improbable due to the insidious onset of the swelling, the patient’s stable systemic status, and the absence of pyrexia, lymphadenopathy, or leukocytosis. Furthermore, tertiary syphilis, scrofuloderma, and various cervicofacial malignancies were histopathologically excluded. The lack of regional lymphadenopathy further corroborated a diagnosis of actinomycosis, as the significant morphological dimensions of the pathogen typically preclude lymphatic dissemination. Consequently, actinomycosis should remain a primary diagnostic consideration for all soft tissue swellings of the head and neck [20].

According to a study by Brook et al., when diagnosing cervicofacial actinomycosis, one should also consider the following diagnosis: abscess by other typical bacteria, cyst, neoplasm, tuberculosis, or nocardiosis [20]. *Nocardia* species are partially acid-fast and typically demonstrate positive staining with modified acid-fast techniques, whereas *Actinomyces* species are not acid-fast, which aids in differentiating these organisms microscopically. Similarly, botryomycosis may present with a comparable clinical morphology, but its granules are characterized by the presence of non-filamentous cocci rather than filamentous bacilli [21].

Lymphadenopathy represents a prevalent clinical manifestation across a broad spectrum of neoplastic and infectious etiologies. While infectious lymphadenopathy typically exhibits non-specific histomorphological features, diagnostic architectural patterns are identifiable in only a limited subset of conditions, such as cat-scratch disease, infectious mononucleosis, syphilis, and HIV infection. In contrast, the histopathological presentation of actinomycosis remains remarkably consistent across diverse anatomical sites, characterized by a concentric arrangement consisting of a peripheral zone of granulomatous inflammatory tissue and a central suppurative core [2,4]. Cat-scratch disease, caused by *Bartonella henselae*, may also present with granulomatous inflammation and should be considered in the differential diagnosis. Histopathologically, it is typically characterized by stellate granulomas, and the organisms may be demonstrated using Warthin–Starry staining, immunohistochemistry for *Bartonella henselae*, or molecular methods such as PCR. Furthermore, while the pronounced fibrosis of the lymph node capsule may resemble the architectural changes of syphilitic lymphadenitis, actinomycotic involvement is characterized by a lack of significant plasmacytic infiltration within the capsule. Notably, lymphadenopathy associated with actinomycosis is an infrequent clinical entity that may closely simulate the presentation of a malignancy [16].

From a radiological perspective, ultrasonography often lacks sufficient discriminatory value to distinguish actinomycotic lesions from malignant neoplasms. Conversely, contrast-enhanced computed tomography serves as a more effective diagnostic adjunct, potentially narrowing the differential. The disease is typically characterized by the formation of suppurative abscesses enveloped by a dense rim of granulomatous inflammation [22].

The radiographic manifestations of cervicofacial actinomycosis are sparsely documented in the current literature. On computed tomography, the condition typically presents as a contrast-enhancing soft-tissue mass characterized by a central zone of low attenuation, frequently accompanied by reactive inflammatory infiltration within the contiguous soft tissues [2,3,8]. Invasion of the adjacent soft tissue, including the muscles, can occur. The radiologic finding may be affected by the course of the disease, any previous antibiotic therapy, and the immune status of the host. In the subacute stage, actinomycosis may result in a slowly progressing, infiltrative mass if not properly treated [23].

Magnetic resonance imaging demonstrated intermediate signal intensities on both T1- and T2-weighted sequences, accompanied by moderate contrast enhancement. These radiological signatures are likely attributable to the underlying histopathological architecture, specifically the extensive granulation tissue and dense fibrovascular proliferation characteristic of actinomycotic lesions [24].

Scientific literature regarding the utility of FDG PET/CT in the evaluation of actinomycosis remains sparse. In a recent retrospective cohort study by Bonnefond et al. involving 28 patients, neoplastic and alternative infectious etiologies were the primary provisional diagnoses. Despite diagnostic imaging being utilized in 82% of the cohort, only a single patient underwent FDG PET/CT examination, highlighting its infrequent application in this clinical context [25].

The diagnostic imaging strategy was directly influenced by the patient’s gestational status and a careful risk-benefit assessment. While CT or MRI are typically employed to define the deep extension of neck masses, ionizing radiation was avoided to minimize fetal exposure. Ultrasonography provided sufficient evidence of the multi-loculated collection and its communication with the skin, allowing for immediate surgical intervention. The absence of deep cervical lymphadenopathy on ultrasound further supported the clinical suspicion of actinomycosis, as this pathogen characteristically respects anatomical barriers and rarely spreads via the lymphatic system.

The clinical presentation often simulates a tumor origin, especially in the absence of suppurative and granulomatous character, which may be the subject of unnecessary investigations and procedures and delayed treatment [26].

The clinical presentation of cervicofacial actinomycosis often overlaps with that of malignant neoplasms or chronic infections, including tuberculosis and fungal etiologies. A significant clue for differentiating actinomycosis from malignancy is the lack of lymphatic involvement despite the presence of a substantial, infiltrative mass. In contrast, infectious conditions such as tuberculosis or coccidioidomycosis are frequently characterized by the development of matted cervical adenopathy, which is a feature notably absent in most actinomycotic cases [24].

A notable clinical feature in this case was the complete absence of systemic symptoms, such as fever, chills, or weight loss, despite the presence of multiple fistulous tracts and a four-month disease progression. This indolent presentation is a hallmark of cervicofacial actinomycosis, which often mimics slow-growing neoplasms rather than acute pyogenic infections. In the context of pregnancy, this lack of systemic inflammatory response can further contribute to diagnostic delays, as seen with the initial misdiagnosis of a simple cutaneous furuncle.

Although cervicofacial actinomycosis occurs infrequently, it should be included in the differential diagnosis when images show a soft-tissue mass with inflammatory changes and an infiltrative nature in the cervicofacial area.

When considering the antimicrobial therapy of *Actinomyces* infections, clinicians should be aware of the antibiotics having anti-anaerobe activity. The first-line therapy of actinomycosis is high-dose therapy with intravenous penicillin G (12–24 million U/day for adults) or ampicillin for 2–6 weeks, which should be replaced in a sequential fashion (and if clinical improvement is observed) by penicillin V or amoxicillin per os for an extended period of time (6–12 months) to prevent relapse [2].

Owing to the extensive fibrosis and central necrosis associated with actinomycotic lesions, the affected tissue becomes hypovascular, thereby limiting the pharmacological bioavailability of antibiotics at the infection site. To circumvent this, the primary therapeutic paradigm involves high-dose penicillin administered over an extended duration, ranging from 6 months to a year. However, successful clinical outcomes have been documented with abbreviated treatment durations of less than 6 months, specifically in cases localized to the cervicofacial region [27].

Diagnostic accuracy remains the cornerstone of effective clinical intervention. Regarding actinomycosis, the conventional requirement for a 12-month course of antibiotics is being re-evaluated in favor of personalized regimens adjusted according to the patient’s progress. Surgical intervention is primarily focused on the decompression of fistulous abscesses; however, more invasive resective surgery is indicated only in the presence of widespread necrotic lesions or when the infection fails to respond to pharmacological treatment [13].

Actinomycosis remains underdiagnosed for several reasons, including failure to communicate clinical suspicion to the microbiology laboratory and the difficulty of obtaining representative specimens from heterogeneous purulent material. In the present case, three aspirated samples were obtained; however, the organism was recovered from only one culture. Careful microscopic examination of direct smears remains essential, as the presence of filamentous, branching Gram-positive rods with a beaded appearance strongly supports the diagnosis of actinomycosis even when culture recovery is limited.

Penicillin or amoxicillin/clavulanic acid are the preferred antibiotic regimens found in the literature [28].

Large studies on cervicofacial actinomycosis are lacking. Therefore proper guidelines for treatment and treatment duration are difficult to establish.

In the present case, diagnosis was particularly challenging because the patient presented with a chronic, fistulized cervical mass initially interpreted as a benign cutaneous infection, resulting in delayed referral. Pregnancy further limited the use of invasive diagnostic procedures and required careful selection of therapeutic options. The presence of filamentous, branching Gram-positive rods with a beaded appearance on Gram staining strongly supported the diagnosis of actinomycosis, while microbiological identification of *Actinomyces georgiae* provided additional confirmation and guided targeted antibiotic therapy. Conservative surgical management combined with appropriate antimicrobial treatment led to progressive resolution of the lesions, while the pregnancy evolved normally. The favorable outcome highlights the importance of early suspicion and close collaboration between otorhinolaryngology, infectious diseases, and obstetrics teams.

This case has some limitations. Gram staining demonstrated elongated, filamentous Gram-positive rods suggestive of actinomycosis, and the organism was subsequently identified as *Actinomyces georgiae* by culture. Although the Gram stain findings were sufficient to strongly support the diagnosis, culture identification provided additional microbiological confirmation.

Although it is a rare and interesting infectious cervical condition, cervicofacial actinomycosis involves some issues regarding the differential diagnosis among neck masses.

This case is particularly distinctive as it represents, to our knowledge, the first documented report of cervicofacial *actinomycosis specifically attributed to Actinomyces georgiae* (recently reclassified as *Schaalia georgiae*) during pregnancy. Furthermore, the upper laterocervical localization and the presence of multiple fistulae without regional lymphadenopathy provide a critical diagnostic reminder: actinomycosis can mimic malignancy or tuberculosis even in atypical anatomical sites. The diagnostic process during pregnancy requires a nuanced approach to imaging; while ionizing radiation (CT or X-ray) is not strictly contraindicated, it was avoided in this case based on a risk-benefit assessment, prioritizing ultrasound and clinical findings due to the unavailability of immediate MRI.

Cervicofacial actinomycosis typically presents in the submandibular region, but our case is distinctive for its left upper laterocervical localization. While species like *A. israelii* are common globally (often associated with pelvic infections in pregnancy), this report represents the first documented case of cervicofacial actinomycosis specifically attributed to *Actinomyces georgiae* in a pregnant patient. The diagnosis of actinomycosis was established through the cytological findings (Gram stain direct diagnosis), and recovery of *A. georgiae* further supported it. Identification of bacterial colonies composed of filamentous, Gram-positive rods with a beaded appearance on Gram stain was done with MALDI-TOF mass spectrometry after 96 h anaerobic incubation. The treatment was optimized for fetal safety, using amoxicillin-clavulanic acid (1 g every 12 h) for 3 months, resulting in complete resolution.

## 4. Conclusions

This case highlights the diagnostic complexity of *Actinomyces georgiae* presenting as a fistulized neck mass during pregnancy. The atypical clinical manifestation and the absence of systemic symptoms necessitated a high index of suspicion and microbiological confirmation via MALDI-TOF MS. Despite the constraints on ionizing radiation imaging during gestation, a multidisciplinary approach ensured a successful clinical outcome. Long-term therapy with amoxicillin-clavulanic acid proved to be both safe for fetal development and effective for maternal recovery. Ultimately, this report contributes to the clinical literature by expanding the known manifestations of *A. georgiae* in the context of pregnancy.

## Figures and Tables

**Table 1:** Altered values of the complete blood count and biochemistry parameters.

Parameter	Value	Refference Value
Hemoglobin	11.8 g/dL	12.0–15.5 g/dL
Haematocrit	34.8%	35.0–45.0%
Alkaline reserve	18.8 mmol/L	22–29 mmol/L
Urea	10 mg/dL	16.6–48.5 mg/dL
Direct bilirubin	0.37 mg/dL	0.01–0.30 mg/dL
Total bilirubin	1.26 mg/dL	0.146–1.20 mg/dL

## Data Availability

All relevant data are included in the article. Patient consent was obtained in accordance with ethical standards.
